# Protease Inhibitors and Innate Immune Agonists as Antiviral Strategies Against Dengue and Zika Viruses

**DOI:** 10.3390/pathogens15020232

**Published:** 2026-02-19

**Authors:** Marianna Costa, Paola Trischitta, Federica Mastrolembo Barnà, Maria Teresa Sciortino, Rosamaria Pennisi

**Affiliations:** 1 Department of Chemical, Biological, Pharmaceutical and Environmental Science, University of Messina, Viale Ferdinando Stagno d’Alcontres 31, 98166 Messina, Italy; marianna.costa1@studenti.unime.it (M.C.); paola.trischitta@studenti.unime.it (P.T.); federica.mastrolembobarna@unime.it (F.M.B.); mtsciortino@unime.it (M.T.S.); 2 Department of Chemistry, Biology, and Biotechnology, University of Perugia, Via Elce di Sotto 8, 06123 Perugia, Italy

**Keywords:** protease inhibitors, antivirals, flavivirus, innate immune agonists

## Abstract

Emerging mosquito-borne flaviviruses, such as Dengue virus (DENV) and Zika virus (ZIKV), pose major global public health threats due to their geographic expansion, climate change, and the absence of effective antiviral therapies. Antiviral development against these pathogens has primarily focused on two complementary strategies. On the one hand, the blocking of viral replication by directly inhibiting essential viral enzymes, and on the other, enhancing the host’s innate immune defenses via targeted activation of intracellular antiviral pathways. Among the viral proteins required for replication, the NS2B–NS3 protease complex is one of the most conserved and druggable targets, prompting extensive efforts to design both covalent and non-covalent inhibitors. Covalent inhibitors, such as boronic acids, aldehydes, trifluoromethyl ketones, phenoxymethylphenyl derivatives, and α-ketoamides, form irreversible or slowly reversible bonds with the catalytic serine residue (Ser 135), producing long-lasting and high-affinity suppression of protease activity. In parallel, several classes of non-covalent, particularly allosteric, inhibitors have emerged as promising alternatives with improved specificity and reduced off-target reactivity. A complementary antiviral strategy involves the use of agonists of key innate immune sensors such as TLRs, RIG-I, and the cGAS–STING axis, which mediate the release of interferons (IFNs). This review brings together current knowledge on these two mechanistically distinct yet convergent approaches, highlighting how both can ultimately restrict flavivirus replication. Future opportunities involving modified peptide scaffolds, advanced delivery systems, and drug-repurposing strategies are finally discussed for the development of next-generation therapeutics against DENV and ZIKV.

## 1. Introduction

Flaviviruses are a genus of positive-sense, single-stranded RNA (ssRNA) viruses within the *Flaviviridae* family, which includes several relevant human pathogens responsible for outbreaks and severe disease [1]. Many flaviviruses are transmitted by arthropod vectors and are therefore classified as arthropod-borne (arboviruses), such as Dengue virus (DENV), Zika virus (ZIKV), Yellow Fever virus (YFV), and West Nile virus (WNV) [2,3]. Transmission to humans usually occurs through the bite of infected mosquitoes, including *Aedes aegypti*, *Aedes albopictus*, and *Culex* species, predominantly distributed in tropical and subtropical regions [3]. Environmental factors, including temperature, rainfall, and humidity, greatly affect mosquito ecology and virus transmission. Studies conducted in regions like Barbados and Puerto Rico have reported links between climate variability and DENV incidence [4,5]. Broader climate trends may also facilitate the geographic spread of mosquito vectors, potentially allowing flaviviruses to enter regions where they were not previously common [6]. Although ZIKV was less prevalent than DENV in the past, it became a major global health concern during the 2015–2016 epidemic, which was linked to severe neurological complications such as Guillain–Barré syndrome [7]. Despite the public health impact of DENV and ZIKV, no fully effective antivirals are currently approved for either infection. Currently, two live-attenuated dengue vaccines, Dengvaxia^®^ (CYD-TDV, Sanofi Pasteur, Paris, France) and Qdenga^®^ (TAK-003, Takeda, Osaka, Japan), have been approved in several dengue-endemic countries [8,9,10,11]. Additionally, multiple vaccine candidates, including TV-003/TV-005 (Butantan-DV), are in preclinical or early clinical stages of development [12]. However, dengue vaccine development remains a challenge because it is difficult to produce balanced and durable immunity against all four DENV serotypes (DENV-1 to DENV-4) [13]. CYD-TDV (Dengvaxia^®^), the first dengue vaccine licensed for clinical use in individuals aged 9–44 years [14], is a tetravalent live-attenuated chimeric vaccine which comprises the prM/E proteins of the four DENV serotypes (DENV1, DENV2, DENV3, and DENV4) and the non-structural (NS) and capsid proteins of the attenuated yellow fever (YF) 17D vaccine virus [15]. Initially, in 2016, the World Health Organization (WHO) recommended Dengvaxia^®^ only for use in areas with high dengue prevalence, where at least 70% of the target age group had been previously infected [16]. Clinical trials reported an overall vaccine efficacy against DENV-3 and DENV-4, but substantially lower protection against DENV-1 and DENV-2 [8,9,10,17]. However, subsequent long-term follow-up data revealed a critical safety concern. In fact, seronegative individuals were at increased risk of severe dengue if infected after vaccination. This observation led WHO to revise its recommendations in 2018, restricting the use of Dengvaxia^®^ to individuals with confirmed dengue infection [18]. In the Philippines, the vaccination of seronegative children was associated with fatal outcomes, ultimately resulting in the revocation of Dengvaxia’s license and severe social, legal, and political repercussions [19]. TAK-003 (Qdenga^®^) is a second-generation tetravalent live-attenuated dengue vaccine that uses the attenuated DENV-2 PDK-53 strain as a backbone, conferring strong immunogenicity against DENV-2 [20,21,22]. Preclinical studies demonstrated protection against all four DENV serotypes in non-human primates, with acceptable safety profiles [20,23]. The vaccine has been approved by the EMA (European Medicines Agency) for individuals aged ≥ 4 years and is administered as a two-dose regimen [24]. Despite its overall effectiveness, TAK-003 exhibits uneven serotype-specific protection. Vaccine efficacy remains consistently highest against DENV-2 but is notably lower and more variable against DENV-3 and DENV-4, especially in dengue-naïve individuals [11,25,26].

Therefore, although significant progress has been achieved in vaccine development, the treatments currently available have important limitations, highlighting the need for alternative or supplementary strategies to control flavivirus infections.

In this scenario, this review focused on two complementary approaches for developing antivirals against flaviviruses. The first approach aims to directly inhibit viral replication by targeting essential viral proteases with covalent and non-covalent inhibitors that target the NS2B–NS3 protease complex. The second strategy focuses on boosting host antiviral defenses by activating innate immune pathways and intracellular antiviral responses.

## 2. Covalent Inhibitors of NS2B–NS3 Protease

The flavivirus genome is a positive-sense, single-stranded RNA of about 11 kb, consisting of a 5′ untranslated region (UTR), a single long open reading frame (ORF), and a 3′ UTR. The ORF encodes a polyprotein (~3430 amino acids) that is processed both co- and post-translationally by viral and host proteases into three structural proteins (capsid [C], premembrane/membrane [prM/M], and envelope [E]) and seven nonstructural (NS) proteins (NS1, NS2A, NS2B, NS3, NS4A, NS4B, NS5) [27]. Structural proteins mediate viral entry, fusion, and infection of host cells, with the E protein undergoing conformational changes during fusion and prM protecting immature virions. NS proteins are mostly enzymatic but also play key roles in immune evasion. NS1 participates in negative-strand RNA synthesis; NS2A, NS4A, and NS4B mediate membrane rearrangements and interfere with host immune responses; NS2B acts as a cofactor for the NS3 protease; NS3 exhibits protease, helicase, nucleoside-triphosphatase (NTPase), and RNA triphosphatase activities; and NS5 functions as RNA-dependent RNA polymerase, methyltransferase, and guanylyl transferase, while also modulating host immunity [28]. The NS2B–NS3 protease complex is crucial for viral polyprotein processing and flavivirus replication, making it one of the most conserved and druggable targets and driving extensive efforts to develop both covalent and non-covalent inhibitors. Structural studies have demonstrated that the NS2B–NS3 protease undergoes essential conformational changes necessary for catalysis. It shifts between an inactive “open” conformation, where the C-terminal segment of NS2B is disengaged, and an active “closed” conformation, where NS2B tightly associates with NS3. The binding of substrate or inhibitor triggers this closed state, aligning the catalytic triad (Ser–His–Asp) and stabilizing the substrate-binding pockets. A beta-hairpin formed by NS2B in the closed state further shapes the S2 and S3 substrate pockets. This conformational switch is vital for enzymatic function and strengthens the draggability of the NS2B–NS3 protease as an antiviral target [29]. Covalent inhibitors offer a highly effective therapeutic approach. They initially form a reversible complex that positions a reactive chemical group near nucleophilic residues, like cysteine or serine. This facilitates the formation of a covalent bond, leading to long-lasting enzyme inhibition, reduced dosing requirements, and enhanced selectivity, while requiring careful control of reactivity to prevent excessive off-target binding and undesirable side effects [30]. Several classes of covalent inhibitors targeting flavivirus NS2B–NS3 proteases are identified (see Table 1). Peptide-based inhibitors incorporate reactive warheads, such as boronic acid derivatives, aldehyde derivatives, and trifluoromethyl ketones (TFMK), which create covalent bonds with the catalytic serine. Conversely, non-peptidic inhibitors, including phenoxymethylphenyl derivatives and α-ketoamides, provide broader chemical diversity and often have better stability and pharmacokinetic profiles.

### 2.1. Peptide-Based Inhibitors

**Figure 1:**
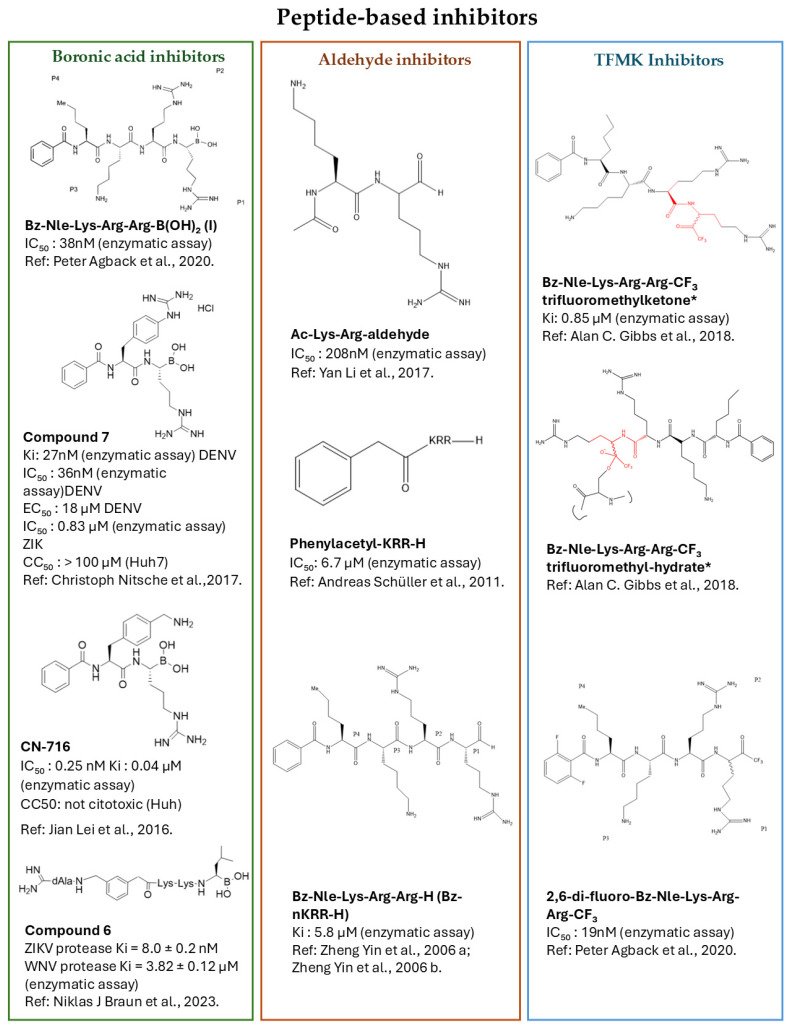
Peptide-based covalent inhibitors of the flaviviral NS2B–NS3 protease. Chemical structures of peptide-derived inhibitors targeting the NS2B–NS3 protease are classified based on their reactive warheads: boronic acid inhibitors (green) [33,34,35,36], aldehyde inhibitors (orange) [30,31,32,33,34,35,36,37,40,41], and trifluoromethyl ketone (TFMK) inhibitors (blue) [33,42]. For each class, selected compounds are shown together with their corresponding enzymatic and cellular activity data, as reported in the literature. Enzymatic inhibition is expressed as IC_50_ (half-maximal inhibitory concentration) or Kᵢ (inhibition constant) values against the NS2B–NS3 protease, whereas antiviral efficacy in cell-based assays is reported as EC_50_ (half-maximal effective concentration) values when available. * Red-highlighted regions show the equilibrium between the trifluoromethyl ketone and hydrate forms in solution.(Chemical structures were drawn using ChemDraw Pro 12.0 CambridgeSoft Corporation, Cambridge, MA, USA).

Peptide-based covalent inhibitors are among the most-studied compounds targeting flavivirus NS2B–NS3 proteases. They usually bind to the enzyme’s active site and form a covalent bond with the catalytic serine residue. This interaction directly engages the catalytic triad and stabilizes a transition-state-like conformation during proteolysis [29]. To improve both potency and stability, various chemical modifications have been applied, especially at the C-terminal region of peptide scaffolds. These modifications include the incorporation of reactive warheads such as boronic acids, aldehydes, and TFMK, as well as the introduction of cyclic constraints (Figure 1, Table 1). Collectively, these strategies improve the binding affinity and proteolytic stability, reduce susceptibility to degradation, and enhance the overall drug-like properties of these inhibitors [31,32].

#### 2.1.1. Boronic Acid Inhibitors

Boronic acid-based inhibitors have emerged as some of the most potent covalent modulators of flavivirus NS2B-NS3 proteases, mainly because of their ability to mimic the transition state of peptide bond hydrolysis. The boronic warhead reacts with the catalytic Ser135 residue, forming a tetrahedral boronate intermediate that stabilizes the enzyme in a transition-state-like configuration. This mechanism explains the high binding affinity and favorable selectivity profiles observed for this class of inhibitors, positioning boronic peptides as valuable scaffolds for antiviral drug development against DENV and ZIKV. Early studies focused on short peptidic boronic acids, including the tetrapeptide Bz-Nle-Lys-Arg-Arg-B(OH)_2_ (I), a derivative of the dipeptide Bz(4-CH_2_NH_2_)Phe-Arg-B(OH)_2_], known as compound **7**, CN-716, and a newly identified member of the series of acyclic boroleucine derivatives, designated as compound **6**.

The tetrapeptide Bz-Nle-Lys-Arg-Arg-B(OH)_2_ (I) forms a covalent adduct with the NS3 active site and stabilizes the H51–D75–S135 catalytic triad through low-barrier hydrogen bonds (LBHBs) [33]. Mutagenesis of Ser135 and His51 confirmed the essential role of the boronic moiety in binding. The resulting bi–covalent Ser–B and His–B interactions generate a compact active-site geometry that explains the inhibitor’s high affinity and supports its relevance as a targeted DENV-2 NS3 scaffold [33]. Further studies on boronic dipeptides confirmed their ability to form reversible covalent interactions with Ser135. The prototype Bz(4-CH_2_NH_2_)Phe-Arg-B(OH)_2_ served as the basis for creating analogues with modified P2 side chains and C-terminal group. Among these, compounds **4** and **7**, both bearing aromatic P2 substituents, showed the highest potency [34]. Compound **7** displayed a Ki of 27 nM against the DENV protease, comparable to the tetrapeptide Bz-Nle-Lys-Arg-Arg-B(OH)_2_ (Ki = 43 nM) and inhibited viral replication without detectable cytotoxicity up to 100 μM, although its high polarity limited cell permeability. Crystallographic data confirmed that the boronic group forms a reversible covalent bond with Ser135, with the tetrahedral boronate oxygen positioned in the oxyanion hole. Binding was further stabilized by P2 hydrogen bonding and π-stacking interactions with His51 and Tyr161, as well as by P1-Arg salt bridges with Asp129 and Tyr130 [34].

The boronic peptide CN-716 also exhibited strong inhibitory potency (IC_50_ = 0.25 μM, Ki = 0.04 μM) [35] due to a reversible covalent bond with Ser135 and the formation of a cyclic diester between the boronic group and glycerol within the S1′ pocket, a region rarely targeted by flaviviral inhibitors. The P1-Arg residue forms a salt bridge with Asp129, while P2 4-aminomethylphenylalanine engages in hydrogen-bonding and ionic interactions with Asp83 and Ser81 of NS2B. CN-716 displayed high selectivity and no detectable cytotoxicity in the human hepatoma cell line (Huh7), supporting its suitability as a lead compound and suggesting that prodrug derivatization could enhance its membrane permeability [35].

Recently, acyclic inhibitors containing boroleucine as a neutral P1 residue were developed to replace the basic guanidinium group of earlier macrocyclic analogues. Among eight synthesized derivatives, compound **6** proved to be the most potent (Ki = 8 nM against ZIKV NS2B–NS3 protease), 200-fold more active than its non-boronic analogue [36]. In contrast, its activity against the WNV protease was significantly lower, indicating high specificity for ZIKV. Crystallographic analysis confirmed a covalent interaction between the P1 boroleucine and Ser135, stabilized within the tetrahedral intermediate by G133, S135, and His51. The P2-Lys side chain forms a salt bridge with Asp83, while the flexible N-terminal region shows weak electron density, suggesting minimal interaction with Asp129. The P3-Lys residue contributes to backbone stabilization through a short antiparallel β-sheet with G151 and G153, orienting the P2–P1 segment optimally for catalytic engagement despite the absence of direct contacts with D129 [36].

#### 2.1.2. Aldehyde Inhibitors

Aldehyde-based peptide inhibitors represent one of the earliest and most extensively characterized classes of reversible covalent compounds targeting flaviviral NS2B–NS3 proteases. Their mechanism depends on the aldehyde warhead reacting with the catalytic Ser135, forming a reversible hemiacetal adduct that keeps the protease inactive.

Crystallographic and NMR analyses of simple dipeptides, such as Ac-Lys-Arg-aldehyde, revealed a strong binding to the ZIKV protease (IC_50_ = 208 nM), due to the interaction with Asp75, Asp129, Tyr130, Tyr161, Asn152, and Ser81 which stabilize the NS2B–NS3 closed state [37]. Nucleophilic attack of Ser135 on the aldehyde carbon, coupled with proton transfer to His51, is central to maintaining the inactive closed enzyme conformation [37].

Although these studies firmly established aldehydes as effective transition-state mimics, their utility has remained largely limited to structural characterization rather than advancing into translational drug development.

Extension of this strategy to DENV proteases led to the development of substrate-mimetic tripeptide aldehydes as phenylacetyl-KRR-H, which inhibited DENV protease activity in the low micromolar range (IC_50_ = 6.7 µM) through strong electrostatic complementarity of P2-Arg residue with the S2 pocket [38]. However, comparative structural studies revealed that the DENV S2 pocket is narrower and conformationally more flexible than in other flaviviruses, resulting in pronounced sensitivity to side-chain size and orientation. This structural plasticity adds an element of unpredictability that makes structure-guided optimization more difficult and partly explains why the potency gains in this series are limited [38]. Crystallographic analysis of DENV-3 NS2B–NS3 protease complexed with the tetrapeptide Bz-nKRR-H (benzoyl-norleucine-Lys-Arg-Arg-aldehyde) highlights both the advantages and intrinsic limitations of this approach. Binding involves the inducible formation of a β-hairpin in NS2B that surrounds NS3, along with the creation of a covalent hemiacetal with Ser135 and stabilization of the oxyanion hole (Gly133–Thr134–Ser135) [39]. The P1-Arg residue stabilizes binding through interactions with Asp129 and Phe130, and P2/P3 residues engage Gly82, Met84, Gly151, and Asn152. However, inhibitor binding is coupled to an entropically unfavorable conformational rearrangement, which limits achievable affinity and contrasts with more readily accessible closed conformations observed in related flaviviral proteases such as WNV. An additional allosteric pocket was found on the opposite side of the catalytic site, which could potentially be targeted by non-covalent ligands that stabilize the closed conformation [39].

Parallel studies on the same substrate-derived tetrapeptide (benzoyl-norleucine-Lys-Arg-Arg) enabled detailed mapping of the active-site functional requirements and comparison of different electrophilic warheads. Aldehydes (benzoyl-norleucine-Lys-Arg-Arg-aldehyde) emerged as reversible, competitive inhibitors (Ki = 5.8 µM), while other warheads, such as α-ketoamides or keto-heterocycles, were unstable or weakly active [40,41]. Structure-activity relationship analyses (SAR) showed that P2-Arg is the key determinant of affinity, P1 can be replaced with neutral aromatic residues (Phe, p-Me-Phe, Trp) without a significant loss of potency, and P4 contributes marginally. Truncation to tripeptides (Ki = 1.5 µM) or even dipeptides (Ki = 12 µM) preserved inhibitory activity and improved drug-likeness.

#### 2.1.3. Trifluoromethyl Ketone Inhibitors

TFMK inhibitors are among the most prominent strategies for targeting the DENV NS2B–NS3 protease through a covalent yet reversible mechanism. These molecules are designed as substrate-mimetic peptides in which the reactive -COCF_3_ warhead substitutes the natural C-terminal carboxylate. Upon binding, the warhead reacts with the catalytic Ser135, forming a stable hemiketal intermediate that closely resembles the transition state of proteolytic cleavage. As a result, the protease becomes trapped in an inactive conformation, blocking viral polyprotein maturation.

The prototype inhibitor Bz-Nle-Lys-Arg-Arg-CF_3_ has been shown to bind the DENV-2 NS2B–NS3 protease with submicromolar affinity (Ki_ii ≈ 0.85 μM). Replacing the TFMK warhead with a nonreactive group caused a dramatic loss of potency, with Kᵢ values exceeding 500 μM [42]. NMR analyses further showed that Bz-Nle-Lys-Arg-Arg-CF_3_ does not adopt a single pose within the active site but instead alternates between two dynamic binding modes. In one configuration, the N-terminal region of the inhibitor inserts into the structural β-barrel 1 domain of the NS3 (trifluoromethylketone), and then extends along β-barrel 2, resulting in a conformation that enhances solubility (trifluoromethyl-hydrate). In both binding models, the C-terminal Arg-Arg dipeptide forms electrostatic interactions with acidic residues Glu91–Glu93 of NS2B, which serve as key anchoring points. These dynamic binding modes enhance the reciprocal conformational flexibility of both the inhibitor and the enzyme, supporting binding stability and overall inhibitory efficiency [42].

A subsequent study further examined TFMK-based inhibitors using an “unlinked” NS2B–NS3 protease construct, which enabled more detailed characterization of active-site dynamics. Within this series, the 2,6-di-fluoro-Bz-Nle-Lys-Arg-Arg-CF_3_ analogue proved to be particularly promising due to its ability to form a reversible covalent bond with Ser135 and to stabilize a tetrahedral intermediate through interactions with the oxyanion hole (Gly133 and Ser135) [33]. NMR analyses confirmed that the TFMK warhead is positioned within a largely hydrophobic microenvironment with limited water accessibility. This environment favors the formation of an LBHB between His51 and Asp75, thereby reinforcing the stability of the catalytic triad and contributing to the overall inhibitory effect.

### 2.2. Non-Peptidic Inhibitors

**Figure 2:**
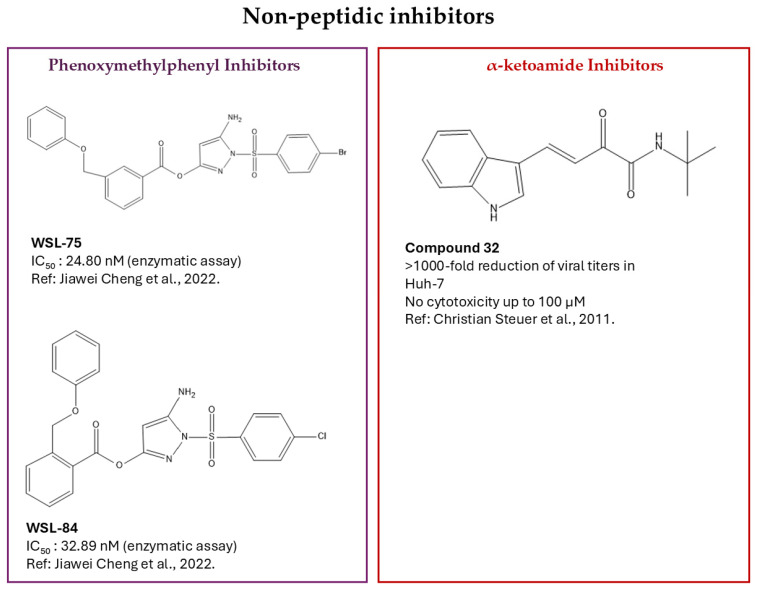
Non-peptidic inhibitors of the flaviviral NS2B–NS3 protease. Chemical structures of non-peptidic small-molecule inhibitors targeting the NS2B–NS3 protease are shown, including phenoxymethylphenyl derivatives (purple) and α-ketoamide inhibitors (red) [43,44]. For each class, chemical structures are shown together with their corresponding enzymatic and cellular activity data, as reported in the literature. Enzymatic inhibition is expressed as IC_50_ (half-maximal inhibitory concentration). (Chemical structures were drawn using ChemDraw Pro 12.0 CambridgeSoft Corporation, Cambridge, MA, USA).

Alongside the development of covalent peptide-based inhibitors for the flavivirus protease, there has been increasing focus on non-peptidic small molecules. Traditional inhibitor design has mainly used tetrapeptides or longer chains that imitate the non-prime residues of the natural substrate and include electrophilic groups to target the catalytic serine. However, these peptide frameworks are naturally limited by their high basicity, primarily due to Arg and Lys residues, and by their large size, which hampers cell permeability and diminishes their therapeutic potential. To overcome these limitations, attention has increasingly shifted toward non-peptidic scaffolds that maintain strong reactivity toward the catalytic serine while offering physicochemical properties more suitable for small-molecule antiviral drugs. In this context, small electrophilic molecules, such as phenoxymethylphenyl derivatives and α-ketoamides, are a promising alternative (Figure 2, Table 1). These inhibitors use a reversible covalent mechanism similar to peptide-based agents but benefit from reduced size and enhanced cell permeability [43].

#### 2.2.1. Phenoxymethylphenyl Derivatives

An additional advancement in the development of covalent inhibitors of the DENV NS2B–NS3 protease was achieved by introducing phenoxymethylphenyl substituents into the previously identified pyrazole ester scaffold. This modification was made to improve both binding stability and antiviral effectiveness by strengthening hydrophobic and π–π interactions within the active site. WSL-75 and WSL-84 are analogues of WSL-01 (IC_50_ = 129 nM) modified by the introduction of a phenoxymethylphenyl group at a strategically selected position [44]. These new derivatives exhibited a marked increase in inhibitory activity, with IC_50_ values of 24.8 nM and 32.9 nM, respectively. Enzymatic and computational studies confirmed an ester-type covalent mechanism, in which the pyrazole warhead forms a covalent bond with Ser135 of the catalytic triad. Covalent docking and molecular dynamics (MD)simulations further revealed a network of additional stabilizing interactions, including hydrogen bonds with Gly133 and Val36, as well as π–π stacking with Tyr161 in the case of WSL-75 and with Tyr150/His51 for WSL-84. Notably, WSL-84 also established a halogen bond with Gln27, consistent with the presence of a chlorinated substituent at the R_1_ position. These structural features resulted in a significant antiviral effect in DENV-2-infected baby hamster kidney-21 cells (BHK-21) [44].

#### 2.2.2. α-Ketoamide Inhibitors

Among the emerging classes of covalent inhibitors targeting the DENV virus NS2B–NS3 protease, α-ketoamide derivatives have attracted significant interest for their ability to form reversible covalent adducts with serine proteases.

Steuer and colleagues conducted an extensive screening of small molecules containing a β, γ-unsaturated α-ketoamide moiety, leading to the synthesis of several promising analogues. Among these, compound **32** emerged as the most promising candidate [43] for its potent antiviral activity, selectivity toward the NS2B–NS3 protease, and lack of detectable cytotoxicity [43]. Structurally, compound **32** has an indole group at position 3, which promotes binding to the enzyme’s active site. The indole-NH group serves as a hydrogen-bond donor, stabilizing interactions with Tyr150 and Asp129 in the S1 catalytic pocket. The process involves the catalytic Ser135 nucleophilically attacking the α-ketoamide carbonyl, forming a reversible covalent hemiketal intermediate. Functionally, compound **32** demonstrated potent in vitro antiviral activity against DENV-2 in Huh-7 cells, reducing viral titer by more than three orders of magnitude compared to the control, with the effect being strictly dose-dependent [43].

### 2.3. Strengths, Limitations, and Translational Barriers of Covalent NS2B–NS3 Protease Inhibition

Covalent inhibition of the flaviviral NS2B–NS3 protease has been explored using two main strategies: peptide-derived inhibitors and non-peptidic small-molecule inhibitors. Both classes rely on electrophilic groups that react with the catalytic Ser135 to block viral polyprotein processing, yet they differ significantly in how readily they can be developed into antiviral drugs.

Peptide-based covalent inhibitors, such as boronic acids, aldehydes, and trifluoromethyl ketones, have demonstrated strong enzymatic potency and have been essential for uncovering key structural and dynamic features of the NS2B–NS3 protease. Boronic acid derivatives like Bz-Nle-Lys-Arg-Arg-B(OH)_2_, CN-716, and the boroleucine-based compound **6** form reversible covalent adducts with Ser135 and stabilize the catalytic triad through low-barrier hydrogen bonds [33,34,35,36]. Similarly, aldehyde inhibitors like Ac-Lys-Arg-aldehyde and Bz-nKRR-H efficiently trap the protease in its closed, inactive state by forming hemiacetal intermediates, allowing detailed crystallographic and NMR analysis of substrate recognition and NS2B ordering [37,38,39,40,41]. Despite these strengths, peptide-based covalent inhibitors are consistently hampered by profound translational limitations. Their highly peptidic nature, high basicity due to multiple Arg and Lys residues, and overall polarity severely limit membrane permeability and systemic exposure [34,35]. Additionally, the chemical instability of aldehydes [37,38,39,40,41], the buffer- and stereochemistry-dependent behavior of TFMK warheads [33,42], and the intrinsic reactivity and off-target liability of boronic acids [34,35] further complicate pharmacokinetic predictability and safety. Even highly optimized compounds, like compound **6**, despite achieving exceptional enzymatic potency, remain poorly suited for in vivo translation [36]. As a result, peptide-based inhibitors have largely remained powerful mechanistic probes and structural tools rather than realistic drug candidates. In contrast, non-peptidic covalent inhibitors share the same mechanism of action, but their larger size improves stability and reduces permeability issues. Phenoxymethylphenyl pyrazole esters, such as WSL-75 and WSL-84, form a covalent bond with Ser135 through their ester groups and further strengthen the bond through hydrophobic contacts, π–π stacking, and halogen-bond interactions within the active site [44]. However, both WSL-75 and WSL-84 are highly lipophilic and show very low predicted solubility, raising concerns regarding bioavailability and pharmacokinetics, while their selectivity against host serine proteases remains insufficiently characterized [44]. α-Ketoamides, although chemically more stable and selective, display only moderate enzymatic affinity and require further optimization at the β-position and amide nitrogen to enhance potency and specificity [43]. Moreover, for both inhibitor classes, comprehensive in vivo validation and long-term safety assessment remain translational barriers.

**Table 1:** Covalent inhibitors of NS2B–NS3 protease.

Inhibitor	Type of Inhibitor	Virus Target	Action Site	References
Bz-Nle-Lys-Arg-Arg-B(OH)_2_ (I)	Tetrapeptide–boronic acid derivative inhibitor	DENV-2	Active site (Ser135, catalytic triad)	[33]
Compound **7**	Peptide–boronic acid derivative inhibitor	DENV	Active site (Ser135, catalytic triad)	[34]
CN-716	Boronic peptide	ZIKV	Active site (Ser135, catalytic triad)	[35]
Compound **6**	Boroleucine derivatives inhibitor	ZIKV	Active site (Ser135, catalytic triad)	[36]
Ac-Lys-Arg-al	Dipeptide aldehyde inhibitor	ZIKV	Active site (Ser135, catalytic triad)	[37]
Phenylacetyl-KRR-H	Substrate-mimetic tripeptide aldehydes	DENV	Ser135 of the catalytic triad	[38]
(Bz-nKRR-H) Bz–Nle–Lys–Arg–Arg–aldehyde	Tetrapeptide aldehyde inhibitor	DENV/DENV-3/ ZIKV	Active site (Ser135, catalytic triad)	[40,41]
Bz-Nle-Lys-Arg-Arg-CF_3_	Trifluoromethyl ketone inhibitors	DENV-2	Active site (Ser135, catalytic triad)	[42]
2,6-di-fluoro-Bz-Nle-Lys-Arg-Arg-CF_3_ derivative	Trifluoromethyl ketone inhibitors	DENV-2	Active site (Ser135, catalytic triad)	[33]
WSL-75 and WSL-84	Derived phenoxymethylphenyl inhibitor	DENV-2	Active site (Ser135, catalytic triad)	[44]
Compound **32**	α-ketoamide derivative	DENV-2	Active site (Ser135, catalytic triad)	[43]

## 3. Non-Covalent Inhibitors

**Figure 3:**
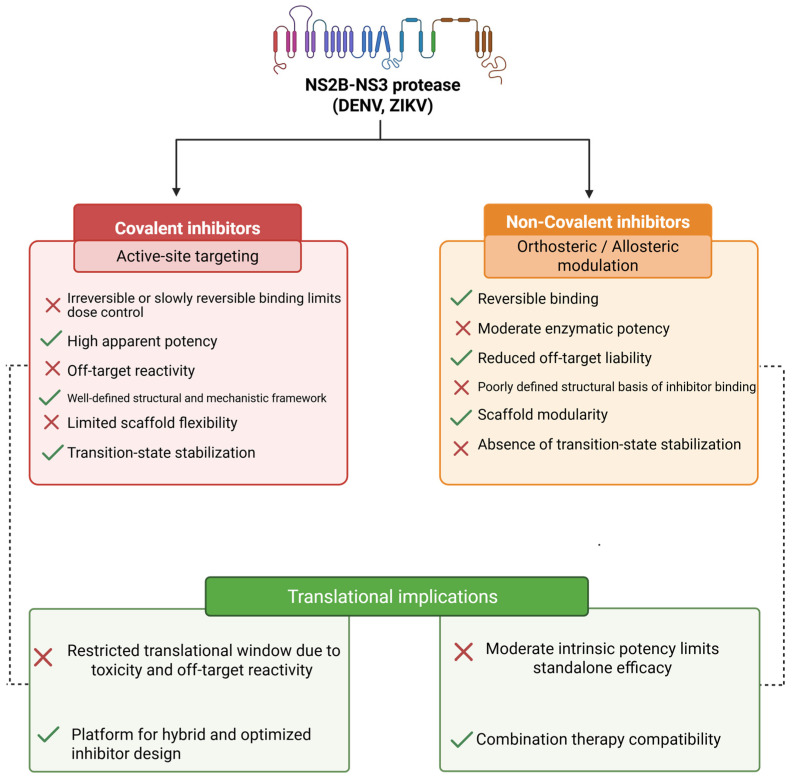
Comparative strengths, limitations, and translational implications of covalent and non-covalent inhibitors targeting the NS2B–NS3 protease. Covalent inhibitors (**left**, red) target the catalytic Ser135 residue via irreversible or slowly reversible binding, which confers high apparent potency but also off-target reactivity, cross-inhibition of host serine proteases, pharmacokinetic liabilities, and a restricted translational window. In contrast, non-covalent inhibitors (**right**, orange) bind reversibly to orthosteric or allosteric pockets, stabilizing the protease’s inactive conformation and reducing toxicity risk. The **bottom** panel (green) highlights the translational implications and trade-offs of covalent and non-covalent inhibition strategies. (Created in https://www.biorender.com).

Following extensive studies on covalent inhibitors, increasing attention has turned to non-covalent inhibitors of the NS2B–NS3 protease. Unlike covalent ligands, which irreversibly bind to the catalytic site, non-covalent molecules (allosteric/orthosteric inhibitors) bind reversibly to regions distal to the active site. These interactions stabilize inactive conformations of the protease and reduce enzymatic activity without competing with high substrate concentration or modifying catalytic residues [45,46]. From a translational standpoint, covalent inhibitors demonstrate high apparent potency and extended target engagement. Nonetheless, their pharmacokinetic profiles can be unfavorable because of electrophilic warheads and peptidic scaffolds [34,35]. Conversely, non-covalent inhibitors do not cause permanent protein modifications, reducing the risk of toxicity and enhancing safety margins [34,35]. Moreover, targeting non-catalytic and conformationally dynamic regions of the protease may reduce the likelihood of resistance development (Figure 3) [47]. The identification of natural and synthetic non-competitive inhibitors further supports allosteric modulation as a viable translational strategy for the development of broad-spectrum antiviral agents [47,48]. Through orthosteric or allosteric binding, non-covalent inhibitors influence catalytic loop dynamics and modulate enzyme turnover. Although lower binding affinities and transient interactions may limit their potency, their reversible nature minimizes cross-reactivity with host proteases. At the same time, the modularity of their scaffolds allows optimization of solubility and permeability. This flexibility supports their use as complementary agents in combination therapies and as potential broad-spectrum antivirals [49].

An auspicious site lies near Ala125, within the 120s–150s loop region. Small molecules binding to this cavity stabilize the inactive open state and prevent the transition to the catalytically competent conformation required for polyprotein processing. The pocket contains hydrophobic residues that enhance affinity with polar ones, which provide specificity, creating a versatile environment for ligand accommodation [48]. Computational studies further identified two conserved allosteric pockets: a transient cavity formed by Ala125, Asp129, Gly133, Tyr150, Gly151, and Asn152, and an open site near the NS2B-binding region involving Trp83, Ile123, Val155, and Leu149. Their conservation across flaviviruses such as DENV and ZIKV highlights their potential as pan-viral targets [50] (Table 2).

### 3.1. Allosteric Inhibitors

Allosteric inhibition targets regions outside the catalytic cleft to regulate protease dynamics. The binding near Ala125 within the 120s–150s loop prevents the formation of the closed, catalytically active conformation and keeps the enzyme in an inactive, open state [48,50]. Curcumin, a polyphenolic compound derived from Curcuma longa, has been identified as a non-competitive allosteric inhibitor of flavivirus NS2B–NS3 proteases [51].

It binds to an allosteric cavity near the Ala125 loop region of DENV-2 protease, disrupting the C-terminal β-hairpin of the NS2B cofactor, specifically the Ser75-Ser79 and Gly82-Ile86 segments, and increases flexibility over residues Ile76-Glu89. As a result, the protease becomes trapped in an open, inactive conformation in which the NS2B cofactor cannot properly align with the catalytic triad, thereby suppressing enzymatic activity.

Myricetin, a dietary flavonoid commonly found in edible plants, inhibits the DENV-2 protease by binding to two distinct allosteric sites [52]. Binding to the first allosteric site destabilizes the NS2B β-hairpin in the active state, while binding to the other site locks the protease in a catalytically silent state. Thus, myricetin can both disrupt the active conformation and stabilize the inactive one, making it the first known small molecule to act as a “dual-mode” allosteric inhibitor of the DENV-2 protease.

Consistently, myricetin, along with other dietary flavonoids such as curcumin, quercetin, rutin, luteolin, and apigenin, has also been reported to inhibit ZIKV NS2B–NS3 protease through non-competitive, allosteric interactions [53]. Notably, myricetin exhibited the highest potency with a Kᵢ of approximately 0.77 µM and an IC_50_ of 1.26 µM, followed by curcumin (Kᵢ ≈ 2.6 µM; IC_50_ ≈ 3.45 µM). Docking analyses indicated these molecules bind in a lateral allosteric pocket at the NS2B–NS3 interface, similar to the cavity described for DENV-2, suggesting a conserved allosteric region across flaviviruses.

De Sousa and colleagues reported the inhibitory activity of several flavanols and their glycosides, including quercitrin, isoquercitrin, myricetin, and quercetin, against DENV [54]. Within the flavonoid group, Agathisflavone acts as a reversible, non-competitive inhibitor of the DENV-2 and DENV-3 proteases, binding to an allosteric site adjacent to the catalytic triad (IC_50_ 15–18 μM).

Additionally, hesperidin (HST), a citrus-derived compound, was identified as a selective inhibitor of ZIKV NS2B–NS3 protease [55].

Docking and MD analyses revealed an allosteric site at the NS2B–NS3 interface, separate from the catalytic pocket. HST forms a stable hydrogen bond with Asn152 and engages in hydrophobic and van der Waals interactions with Val29, Leu31, Phe37, Thr119, Asp121, and Ile124, supporting a non-competitive inhibitory mechanism. Enzymatic assays reported an IC_50_ under 13 µM and a dissociation constant (Kᴰ) of approximately 18 µM, confirming high affinity and selectivity. However, no significant reduction in viral titers was observed in Vero and Vero E6 cells infected with ZIKV, possibly due to limited cellular permeability or efflux [55]. Moreover, naringenin (NAR), the aglycone of naringin, further exemplifies the potential of citrus-derived flavonoids as allosteric inhibitors of ZIKV NS2B-NS3 protease [56]. Docking studies showed that NAR binds to the protease’s allosteric pocket, forming hydrophobic interactions and weak hydrogen bonds with residues at the NS2B-NS3 interface, without engaging the catalytic triad His51–Asp75–Ser135, consistent with a non-competitive mechanism. In various human cell models, such as alveolar epithelial cells A549, human glioblastoma cell line (A172), human hepatoma cell line (Huh7.5), human monocyte-derived dendritic cells (HMDDCs) infected with multiple clinical ZIKV strains, NAR exhibited dose-dependent antiviral activity during and after infection, with an IC_50_ of 58.8 µM, 90% inhibitory concentration of 154.4 µM (IC_90_), 50% cytotoxic concentration of 693.6 µM (CC_50_), and a selectivity index of 11.8 (SI), without virucidal effects [56]. Additional evidence for naturally occurring allosteric modulators of the DENV protease comes from an extensive screening of the NPASS (Natural Product Activity and Species Source) database [57]. Because the protease’s catalytic site is structurally unfavorable for small-molecule binding, the analysis focuses on an adjacent allosteric pocket that can modulate enzymatic activity. From an initial set of 60 natural metabolites filtered by pharmacophore-based screening, five candidates with the most promising docking results emerged: NPC62903, NPC94179, NPC160854, NPC287429, and NPC79715 [57]. These molecules share structural features characteristic of complex natural products, often polyphenolic and in some cases glycosylated, providing numerous hydrogen-bond donors and acceptors that support stable interactions with residues in the allosteric cavity. Among the selected hits, NPC62903 exhibits the most compelling profile. It forms several well-defined interactions, including hydrogen bonds with Glu122, Val147, Lys74, and Gly124, as well as a π- H contact with Ile123, and demonstrates high stability in MD simulations. Its presence tends to increase the overall compactness of the protease and reduce residue mobility, which aligns with an inhibitory allosteric mechanism. The other key compounds (NPC94179, NPC160854 and NPC287429), although with slightly lower binding affinities, still engage with a network of residues that define the allosteric pocket, such as Trp89, Thr120, Gly121, Glu122, Ile123, Gly124, Gly164, Ile165, Ala166 and Gln167, as well as peripheral residues including Lys73, Lys74, Val78, Gly82 and Met84.

Building on these insights, recent studies have focused on developing small synthetic molecules that target the same or similar allosteric sites with improved potency, stability, and pharmacokinetic properties. These efforts primarily focus on the DENV and ZIKV NS2B–NS3 proteases, where the allosteric pocket is conserved across flaviviruses. Among these, quinoxaline-based scaffolds have emerged as promising synthetic analogues that mimic and improve the allosteric inhibition observed with polyphenolic compounds [58]. Zephyr, J., and colleagues identified molecules that disrupt the interaction between NS2B and the NS3 protease, with representative compounds exhibiting IC_50_ values in the 30–70 μM range. SAR studies highlighted the importance of amino substituents on proline-derived side chains and aromatic groups at the 3-position for inhibitory potency. These observations were further supported by alanine-scanning mutagenesis, which identified residues Q74, W83, I123, N152, and V155 as key contributors to ligand binding at the NS2B–NS3 interface [58].

In line with the strategy of improving natural products through minimal structural modification, azaleatin, a C5-methoxylated derivative of quercetin, was also investigated as an allosteric inhibitor of the DENV NS2B–NS3 protease [59]. The introduction of the methoxy group was intended to enhance metabolic stability and oral bioavailability while preserving antiviral activity, as evidenced by an IC_50_ of 38 µg/mL and a concentration-dependent effect comparable to that of quercetin. Kinetic analyses and molecular docking showed that azaleatin behaves as a non-competitive inhibitor, binding to a lateral allosteric pocket, where it establishes a hydrogen bond with Trp89 and multiple hydrophobic contacts with residues such as Val78, Lys73, and Lys74. The methoxy group at C5 contributes to hydrophobic interactions with Lys74, a residue functionally important for catalytic regulation. MD simulations further confirm that the azaleatin-NS2BNS3 protease complex remains stable over a 100 ns trajectory. That binding helps maintain the enzyme in a closed conformation that restricts substrate access to the active site, consistent with an allosteric inhibitory mechanism. Persistent interactions with residues such as Asn152 and Met84 contribute to stabilizing the complex [59].

Finally, a virtual screening of 545 natural and semi-synthetic compounds identified four particularly promising molecules as allosteric inhibitors of the DENV and ZIKV NS2B–NS3 proteases: cpd48_e and cpd50_e (sulfonamide-vinyl sulfone hybrids), cpd48 (sulfonamide-chalcone analogue), and DN071_f (a berberine-cinnamic acid derivative). These compounds do not bind to the catalytic site but occupy a transient allosteric pocket that forms when the enzyme adopts an open conformation. Binding in this region stabilizes the enzyme in an inactive state, preventing the proper positioning of the NS2B cofactor and thereby blocking the proteolytic activity required for viral replication [60].

Van der Waals forces primarily drive protein-ligand interactions, while some compounds, especially cpd48_e and cpd50_e, also exhibit significant electrostatic and hydrogen-bonding contributions. In the DENV protease, the key recognition residues are K74, L149, and N152. In the ZIKV protease, main interactions involve L76, I123, N152, and V155. Among the compounds, cpd50_e forms the highest number of significant hydrogen bonds, whereas DN071_f shows the most favorable pharmacokinetic profile, particularly in terms of gastrointestinal absorption. These findings indicate that sulfonamide-vinyl sulfone hybrids and natural scaffold derivatives such as berberine can effectively stabilize the inactive conformation of DENV and ZIKV NS2B–NS3 proteases, representing promising candidates for the development of novel allosteric antiviral agents [60].

Shenyou Nie and colleagues investigated a new series of non-peptidic inhibitors targeting the Zika virus NS2B-NS3 protease, based on a 2,6-disubstituted indole scaffold [61]. Out of 73 synthesized compounds, compound **66** showed an IC_50_ of 0.32 µM against the ZIKV protease. The compound also inhibited the homologous DENV protease, though with reduced potency in the micromolar range. Antiviral effects were tested in cell-based assays with ZIKV-infected U87 cells. Compound **66** significantly reduced viral replication with an EC_68_ of 1 µM. Additionally, no notable cytotoxicity was observed at concentrations up to 10 µM (CC_50_ > 10 µM), indicating a favorable initial antiviral selectivity profile. Although the molecular mode of binding to the protease has not been defined in detail, enzyme kinetics data indicate a non-competitive mechanism of inhibition, consistent with the possible involvement of a site distinct from the catalytic pocket [61].

### 3.2. Strengths, Limitations, and Translational Barriers of Non-Covalent NS2B–NS3 Protease Inhibition

Allosteric inhibitors of the NS2B–NS3 protease offer several benefits, including the ability to bind to conserved regions outside the catalytic site. This reduces resistance risks and allows for non-competitive mechanisms that keep the enzyme inactive [50,51,52,53,54,55,59,62]. Numerous natural flavonoids (such as curcumin, myricetin, naringenin, and hesperidin) and newly designed synthetic scaffolds have demonstrated strong enzymatic inhibition and, in some cases, antiviral effects in vitro [54,55,56,57,58,60,61,62,63]. Nonetheless, these compounds face limitations, including moderate micromolar potency, poor bioavailability, limited cell permeability, metabolic instability, and common issues associated with polyphenolic natural products. Despite high in vitro affinity, many show little antiviral activity in infected cells [57,58]. Additional barriers to clinical application include incomplete understanding of their binding mechanisms, the need for pharmacokinetic improvements, and the lack of in vivo validation. These challenges highlight the need to develop more stable and selective synthetic analogues to turn these allosteric modulators into effective antiviral therapies [60,61,62,63] (Figure 3).

## 4. The Innate Antiviral Landscape: PRRs, Interferons, and Host-Targeted Therapeutic Approaches

**Figure 4:**
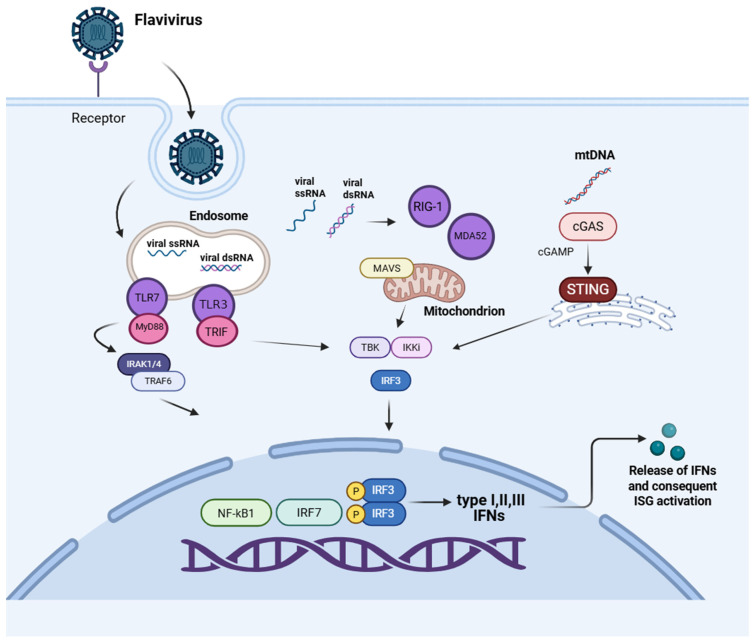
Overview of innate immune pathways that trigger interferon production and activate antiviral ISGs. (Created in https://www.biorender.com).

Although more than 100 antiviral drugs have been approved to date, most act as direct-acting agents that target viral proteins [62]. Despite their effectiveness, a major limitation is the rapid emergence of viral drug-resistant variants. This has led to a focus on host-targeted strategies that strengthen innate immune defenses instead of directly targeting viral components [63]. Innate immunity plays a central role in controlling viral infections (Figure 4). It begins when the pathogen-associated molecular patterns (PAMPs) are detected by pattern recognition receptors (PRRs) [64,65], including Toll-like receptors (TLRs), RIG-I-like receptors (RLRs), NOD-like receptors (NLRs), C-type lectin receptors (CLRs) and the cGAS–STING axis pathway [66,67,68] (Figure 4). Their engagement triggers kinase cascades that activate interferon regulatory factors (IRFs), leading to the induction of type I interferons (IFNs) (IFN-α and IFN-β) and type III (IFN- λ), as well as downstream interferon-stimulated genes (ISGs), that inhibit viral replication and induce an antiviral state in neighboring cells [64,69]. Activation of the interferon response is particularly effective against a wide range of viruses, including emerging pathogens such as ZIKV and DENV [70]. Clinical and preclinical studies have demonstrated that patients infected with DENV exhibit elevated circulating levels of interleukin-2 (IL-2) and IFN-γ [71]. In contrast, in the context of ZIKV infection, studies in human cells have demonstrated robust activation of IFN-α/ϐ, IFN-γ, and IFN-λ responses, accompanied by the induction of numerous ISGs [72]. PRRs are spread throughout the cell. Membrane-associated toll-like receptor 2 and toll-like receptor 4 (TLR2 and TLR4) recognize viral proteins, endosomal toll-like receptor 3,7,8 and 9 (TLR3, TLR7, TLR8, and TLR9) detect viral nucleic acids and cytosolic sensors such as retinoic acid-inducible gene I (RIG-I), melanoma differentiation-associated protein 5 (MDA5), and select NLRs monitor intracellular viral RNA and DNA. The cGAS–STING pathway detects viral DNA in the cytosol by producing cyclic GMP–AMP (cGAMP). This molecule quickly diffuses through the cytoplasm and binds specifically to STING, a transmembrane protein on the endoplasmic reticulum (ER). When cGAMP binds, STING undergoes a conformational change that reveals binding sites for two crucial mediators of antiviral signaling: TBK1, a serine/threonine kinase from the IκB kinase family, and IRF3, a phosphoprotein whose activity is controlled through phosphorylation [73]. The interaction with STING promotes TBK1 autophosphorylation [74], followed by phosphorylation of IRF3, which then dimerizes and translocates to the nucleus, where it induces the transcription of IFNs [75,76]. Once secreted, IFNs bind to their respective receptors and trigger signaling pathways that induce an antiviral state. IFN-α/β bind to interferon-alpha/beta receptors 1 and 2 (IFNAR1 and IFNAR2) and activate the Janus kinase–signal transducer and activator of transcription pathway (JAK–STAT), in which Janus kinase 1 (JAK1) and tyrosine kinase 2 (TYK2) phosphorylate the cytoplasmic transcription factors (STAT1 and STAT2). Phosphorylated STAT1 and STAT2 form a heterodimer that is associated with interferon regulatory factor 9 (IRF9), generating the trimeric complex interferon-Stimulated Gene Factor 3 (ISGF3). ISGF3 then translocates into the nucleus, where it binds interferon-stimulated response elements (ISREs), leading to translation of ISGs [77].

Beyond the canonical JAK–STAT cascade, IFN-I can also trigger alternative STAT-independent pathways, including p38 mitogen-activated protein kinase (p38 MAPK) and Mitogen-Activated Protein Kinase/Extracellular Signal-Regulated Kinase complex (MEK/ERK), which contribute to fine-tuning the transcriptional response [77]. IFN-γ, produced mainly by Natural killer cells (NK) and activated T lymphocytes, binds to the heterodimeric interferon-gamma receptors 1 and 2 (IFNGR1/IFNGR2), leading to the activation of JAK1 and Janus kinase 2 (JAK2). This results in STAT1 phosphorylation, homodimer formation Gamma-Activated Factor (GAF), nuclear translocation, and binding to gamma-activated sequences (GAS) within the promoters of responsive genes [77].

Type III IFNs (IFN-λ1–4) bind to the interferon lambda receptor 1 and interleukin-10 receptor 2 complex (IFNLR1/IL10R2), triggering a signaling pathway like the canonical IFN-I pathway. This involves the formation of ISGF3 (STAT1–STAT2–IRF9) and the induction of a comparable set of ISGs [77]. However, expression of IFNLR1 is primarily restricted to epithelial cells and selected immune cell subsets, making the IFN- λ response more localized and generally less inflammatory than the IFN-I response [78].

Finally, activation of interferon pathways induces more than 300 ISGs [79] which encode proteins with direct antiviral activity, including interferon-stimulated gene 15 (ISG15), Myxovirus resistance protein 1 (Mx1), RNase L, and protein kinase R (PKR) [79]. These effectors block viral transcription, degrade viral RNA, inhibit translation, and modulate protein function involved in protein synthesis [80].

Due to their potent immunomodulatory activity, IFNs have been extensively used as therapies for viral infections and immune-mediated disorders [81]. Since the 1980s, recombinant IFNs have been used to treat chronic viral hepatitis, several types of cancer, and relapsing-remitting multiple sclerosis, with extensive documentation of their efficacy, safety, and tolerability. In most patients, IFN therapy is well tolerated, with predominantly mild, transient adverse effects, such as flu-like symptoms. However, long-term treatment can be associated with more severe adverse events, including leukopenia, thrombocytopenia, hepatotoxicity, and neuropsychiatric effects, sometimes necessitating dose reduction or discontinuation of therapy [82]. This clinical profile suggests that excessive systemic exposure to IFNs may mimic an uncontrolled cytokine release, which is incompatible with the physiological balance of the innate immune response.

In parallel with conventional IFN-based therapies, recent studies have focused on identifying molecules that selectively activate particular components of the innate immune signaling network, including TLRs, the cGAS–STING pathway, and IRF3 (Table 3). These PRR agonists and their effectors mimic infection-driven antiviral responses, promoting IFN and ISG expression while limiting viral replication.

### 4.1. Toll-like Receptor Agonists

Pharmacological activation of TLRs is among the earliest strategies explored to enhance the host’s innate antiviral response. Among these, resiquimod (R848) is a synthetic imidazoquinoline, a class of heterocyclic aromatic compounds. At the molecular level, R848 mimics viral ssRNA and directly engages TLR7 and TLR8 located on endosomal membranes. The interaction with the ligand induces a conformational rearrangement of the receptors that promotes recruitment of the adaptor myeloid differentiation primary response 88 (MyD88), activation of interleukin-1 receptor-associated kinase 1/4 (IRAK1/4) and TNF receptor-associated factor 6 (TRAF6), and phosphorylation of the interferon regulatory factor 7 (IRF7), ultimately leading to the transcription of IFNs and downstream antiviral effectors [83,84,85].

The antiviral efficacy of R848 has been clearly demonstrated against ZIKV and in human monocytes and monocyte-derived macrophages (MDMs) infected with ZIKV. Pretreatment with R848 resulted in a marked reduction in viral RNA levels, with approximately a 30-fold decrease at 1 μM. Transcriptomic profiling revealed the induction of a broad panel of ISGs, with the ISG viperin emerging as a critical mediator of protection [86]. Indeed, genetic knockout of viperin via CRISPR/Cas9 abrogated the antiviral effect of R848, confirming its role downstream of TLR7/8 signaling [87]. Additionally, in vivo studies in Indian rhesus macaques reported the antiviral activity of TLR7/8 agonist CL097M-012 against DENV-1 (Western Pacific 74 strain) [88]. TLR7/8 agonist CL097M-012 (1 mg/animal) was administered subcutaneously on days 2 and 7 after infection, and the treatment during the acute phase resulted in a strong antiviral effect. Viremia was eliminated in three out of four treated animals (75%), while the remaining monkey showed viral levels at the detection limit (25%) and low circulating NS1 protein levels. CL097M-012 modulated the antibody response by decreasing IgG1 levels and increasing IgG2a and IgG2c isotype switching. Additionally, another widely used TLR ligand is polyinosinic:polycytidylic acid [poly(I:C)], a synthetic double-stranded RNA (dsRNA) that exhibits potent antiviral activity against DENV-2 in human hepatoma cells (HepG2), promoting the induction of IFN-I and IFN-III, particularly IFN-β and interleukin-28A/B (IL-28A/B). Notably, the antiviral activity of poly(I:C) was strictly dependent on the timing of administration. Protective effects were observed only under prophylactic or simultaneous treatment conditions, whereas post-infection treatment failed to suppress viral replication [89].

**Table 3:** Immune agonists of interferon-signaling pathways: mechanisms of action and antiviral activity against ZIKV and DENV.

Agonist	Target/ Pathway	Active Concentration	Antiviral Effect	Experimental Model	References
Resiquimod (R848)	TLR7/8→MyD88→ IRF7→IFN-I	1 µM	Suppression of ZIKV replication following pre-treatment.	Human monocytes; monocyte-derived macrophages (MDMs)	[86]
CL097M-012	TLR7/8	1 mg/animal	Marked reduction in DENV viremia during early infection.	Indian rhesus macaques (in vivo)	[88]
Poly (I:C)	TLR3→TRIF→IRF3→ IFN-I/III	5 µg/mL	Potent inhibition of DENV-2 replication (prophylactic or simultaneous treatment conditions).	Human hepatoma cells (HepG2)	[89]
5′pppRNA	RIG-I→MAVS→ TBK1/IKKε→IRF3	1–10 ng/mL	Complete suppression of infectious DENV particle production and blockade of viral RNA synthesis (prophylactic and early therapeutic conditions)	A549 cells, primary human monocytes, and monocyte-derived dendritic cells (MDDCs),	[90]
3p10LG9	RIG-I optimized agonist	EC_50_: U937-DC-SIGN: 3.77 nM; A549: ~0.27 nM; CD11c^+^ DDC: 13.6 nM; LC: 15.5 nM; CD14^+^ DDC: 15.5 nM	Potent, dose-dependent inhibition of DENV replication (prophylactic setting)	U937-DC-SIGN, A549 cells and primary human skin APCs (CD11c^+^ DDCs, CD14^+^ DDCs, LCs)	[91]
M8 dsRNA	RIG-I ligand	A549: 1 ng/mL; Mo-DCs: 10 ng/mL	Potent induction of IFN-β/λ and ISGs with strong inhibition of DENV replication	A549 epithelial cells and Mo-DCs	[92]
DSDP	STING agonist	6.25 μM for DENV; 25 μM for ZIKV	Reduction in intracellular DENV/ZIKV RNA and viral titers	Human fibroblasts (THF)	[93]
diABZI, 2′3′-cGAMP, 3′3′-cGAMP and cAIMP	STING→IRF3	≤100 µg/mL (highest non-toxic dose	Significant inhibition of ZIKV infection	Human foreskin fibroblasts (HFF-1)	[94]
Scleroglucan	Dectin-1→NF-kB	≤100 µg/mL (highest non-toxic dose	Modest anti-ZIKV activity through STING-independent pathway	Human foreskin fibroblasts (HFF-1)	[94]
KIN1400	IRF3 pathway activator	2–20 µM	Reduction in DENV RNA and infectious particle production (active also in the early post-infection window)	Human hepatoma cells (Huh-7)	[95]
AV-C	TRIF→IRF3	IC_90_ of 5.8 μM for ZIKV; IC_90_ of 9.9 μM for DENV	Reduction in ZIKV and DENV replication	Human fibroblasts (THF)	[96]

→ : indicate the downstream signaling pathway activated by each agonist.

### 4.2. RIG-I Agonists

In parallel with TLR agonists, another strategy to pharmacologically enhance innate immunity involves targeting cytosolic receptors that detect viral RNA, such as RIG-I. Among the most extensively studied ligands is 5′-triphosphate RNA (5′pppRNA), a synthetic dsRNA that binds RIG-I, inducing a conformational change and ATP-dependent activation [90]. Activated RIG-I interacts with its mitochondrial antiviral signaling protein adaptor (MAVS) through caspase activation and recruitment domain (CARD–CARD) interactions. MAVS then activates IRF3, IRF7, and Nuclear factor κB (NF-κB) via the IKK-related kinases TBK1 and IKKε, leading to the induction of IFN-β and IFN-α, proinflammatory cytokines, and antiviral genes. Treatment with 5′pppRNA, administered either before or after infection, exerted a strong protective effect in multiple human cell types, including epithelial and myeloid cells. Exposure to concentrations between 1 and 10 ng/mL completely abolished the production of infectious DENV particles, with only a modest reduction in cell viability observed at later time points.

The antiviral effect of 5′pppRNA depends on activation of the RIG-I/MAVS/TBK1/IRF3 pathway but is independent of canonical IFN-I and IFN-III signaling. This suggests RIG-I can directly establish an antiviral state without a strong interferon response. Treatment with 5′pppRNA also causes STAT1 phosphorylation and increases ISGs like IFIT1 and STAT1 [90].

Careful design of dsRNA hairpins has led to the development of a new generation of agonists. The first example is 3p10LG9, a 25-nucleotide dsRNA hairpin based on a previously designed scaffold (3p10L) and, by adding an additional guanosine at position 9, optimized to enhance RIG-I activation [91]. 3p10LG9 triggers more robust IFN-I production than the parental 3p10L construct and, consistently, more strongly enhances ISGs expression. In DENV-permissive cell lines, U937 monocytes expressing DC-SIGN (U937-DC-SIGN) and the human lung carcinoma epithelial cell line (A549), both constructs reduce infection in a dose-dependent manner, but 3p10LG9 is significantly more potent [91]. The antiviral efficacy of 3p10LG9 has been further validated in primary human skin antigen-presenting cells (APCs), a physiologically relevant model for DENV infection. Prophylactic treatment with the immunostimulatory RNA, 3p10LG9, administered 24 h before infection, induces a robust type I interferon response and protects cells in a dose-dependent manner, with EC_50_ values in the low nanomolar range (CD11c^+^ DDCs: 13.6 nM; LCs: 15.5 nM; CD14^+^ DDCs: 15.5 nM) and without evident cytotoxic effects. However, ex vivo studies indicate that 3p10LG9 uptake by skin APCs is minimal in the absence of dedicated delivery systems, highlighting intracellular bioavailability as a critical translational determinant [91].

Engineered variants of 5′pppRNA were generated through gradual modifications of the dsRNA stem [92]. Small changes in stem length and stability were found to influence agonistic potency nonlinearly. In fact, early variants (M1–M4) did not improve activity, whereas extending the stem to 59 nt (construct M5) enhanced IFN-β and interferon-stimulated gene 56 (ISG56) induction and resulted in stronger inhibition of influenza and DENV replication than wild-type 5′pppRNA. Further stem elongation (M6–M8) resulted in progressively higher activity, with M8 emerging as the most potent construct within the series. M8 protects A549 cells from DENV infection at concentrations approximately 100-fold lower than those of the wild-type construct, and in monocyte-derived dendritic cells (Mo-DCs) induces a strong expression of IFN-β, IFN-λ (IL-29), tumor necrosis factor-α (TNF-α), and ISGs such as ISG56, STAT1, and RIG-I. M8 also promotes robust phosphorylation of IRF3 at S396, consistent with activation of the RIG-I, TBK1/IKKε, IRF3 pathway. Knockdown experiments confirm that M8′s activity is strictly RIG-I–dependent, with no cross-activation of MDA5 or TLR3. In RIG-I competent cells, M8 nearly abolishes DENV protein expression, whereas it is inactive in RIG-I–deficient cells [92]. The antiviral activity of M8 is strictly dependent on the timing of administration. Prophylactic treatment establishes a sustained antiviral state, conferring protection for up to 72 h after DENV infection. In contrast, therapeutic efficacy is limited to a narrow early post-infection window, with significant inhibition observed only when M8 is administered within 4 h post-infection. Consistently, immediate post-adsorption treatment reduced the proportion of DENV-infected cells from 33.7% to 9.3%, underscoring timing as a critical determinant of efficacy and supporting the use of RIG-I agonists primarily in prophylactic or very-early therapeutic settings [92].

### 4.3. cGAS–STING Pathway Agonists

In addition to RIG-I agonists, a new class of molecules has been identified to activate the cGAS–STING pathway pharmacologically. Among these, a dispiro-diketopiperazine compound, (DSDP) [2,7,2″,2″-dispiro[indene-1″,3″-dione]-tetrahydrothiazolo [3,2-a:3′,2′-d]pyrazine-5,10(5aH,10aH)-dione], has been characterized as a human STING agonist and cGAS–STING activator [93]. DSDP strongly induces interferon-stimulated gene 54 (ISG54) promoter activity in HepAD38 cells stably engineered to express the cGAS–STING pathway and an ISG54 promoter–driven reporter (HepAD38/cGAS–STING/ISG54 cells), indicating efficient stimulation of the STING → TBK1 → IRF3 axis. In human cells, DSDP induces dose-dependent expression of IFN-β, IL-29, TNF-α, and IL-6. This activity has been observed in both human fibroblasts with extended replicative lifespan (THF cells) and peripheral blood mononuclear cells (PBMCs). However, in PBMCs, the response is skewed toward IFN-I/IFN-III (IFN-β, IL-28A, IL-29) rather than TNF-α. STING activation by DSDP translates into direct antiviral activity: in pretreated THF cells, DSDP reduces DENV and ZIKV replication, with significant suppression of intracellular viral RNA at micromolar concentrations (up to 6.25 μM for DENV and 25 μM for ZIKV) and ≥1-log reductions in viral titers, reaching 2–4 logs at the highest doses. Assessment of in vitro cytotoxicity showed CC_50_ values exceeding 100 μM, indicating that DSDP exerts antiviral effects at concentrations that do not cause overt cytotoxicity. Overall, these data support the existence of a favorable activity window for DSDP in cellular systems; however, the translational relevance of these findings remains to be validated in vivo, given the inherent limitations of in vitro models [93].

Several cyclic dinucleotides (CDNs) have also demonstrated antiviral activity against ZIKV in human foreskin fibroblasts (HFF-1). Compounds such as diABZI, 2′3′-cGAMP, 3′3′-cGAMP, and cAIMP, which mimic second messengers produced by cyclic GMP–AMP synthase (cGAS), reduce ZIKV infection already at the highest non-toxic concentration tested (100 μg/mL). These CDNs rapidly activate the cGAS–STING pathway, promoting phosphorylation of STING (Ser366) and IRF3 (Ser386) within a few hours of treatment, together with NF-κB activation [94].

Interestingly, agonists that do not engage STING can also help restrict viral replication. Scleroglucan, a fungal β-glucan that activates Dectin-1, does not induce STING or IRF3 phosphorylation, yet activates NF-κB and still exhibits antiviral activity against ZIKV, suggesting that alternative, STING-independent pathways can generate a sufficient signal to limit viral replication [94].

Within the small-molecule space, KIN1400 is a synthetic molecule belonging to the family of substituted aminothiazoles. It features an aminothiazole core fused to a benzene ring and, at the scaffold level, resembles a modified hydroxquinoline. KIN1400 represents a third, chemically distinct class of IRF3 activators, sharing no structural similarity with CDN-type STING agonists or RNA-based RIG-I agonists [95]. Treatment of human hepatoma Huh-7 cells with KIN1400 induces expression of MDA5, IFIT1, Mx1, and other ISGs in a dose-dependent manner. KIN1400 exhibits potent antiviral activity against DENV-2 in human hepatoma Huh-7 cells. A concentration of 2 μM is sufficient to achieve >50% reduction in intracellular viral RNA, corresponding to an approximately 4-log decrease in infectious particle production at 24 h post-infection. At higher concentrations (20 μM), KIN1400 retains antiviral efficacy even when administered several hours after infection, with activity comparable to or exceeding that of IFN-β, thereby defining a broader therapeutic window [95].

Finally, a particularly innovative small heterocyclic molecule has recently been identified: 1-(2-fluorophenyl)-2-(5-isopropyl-1,3,4-thiadiazol-2-yl)-1,2-dihydrochromeno[2,3-c]pyrrole-3,9-dione, termed AV-C [96]. This compound combines, in a single structure, a chromene ring fused to a pyrrole, a substituted 1,3,4-thiadiazole moiety, and a fluorinated phenyl group. In human fibroblasts, AV-C promotes IRF3 phosphorylation and subsequent ISG transcription, including ISG15, IFIT1, viperin (RSAD2), and Myxovirus resistance protein 2 (Mx2), and enhances IFN-β production. Functionally, the compound acts upstream of TBK1/IKKε but independently of the adaptors STING and MAVS, instead requiring TIR-domain–containing adapter-inducing interferon-β (TRIF) as a key signaling component. Activation of the TRIF–TBK1/IKKε–IRF3 pathway culminates in IFN-β secretion and engagement of the IFNAR–JAK/STAT circuit, which establishes an autocrine/paracrine antiviral state.

Both ZIKV and DENV show reduced replication in AV-C–pretreated cells, albeit with different sensitivities. ZIKV replication is reduced by ~2.5 logs with an IC_90_ of 5.8 μM and remains susceptible even when AV-C is added up to 16 h post-infection. DENV exhibited lower susceptibility, with an IC_90_ of ~9.9 μM, consistent with its reduced replication in fibroblasts and with differences in the ability of individual flaviviruses to counteract innate antiviral signaling in this cellular context [96].

### 4.4. Translational Challenges: Safety, Dosing, and Timing

Although numerous innate immune agonists display strong antiviral activity in vitro and ex vivo models, their clinical translation as host-directed therapies requires careful optimization of dose, timing of administration, and safety. Agonists targeting TLRs, RIG-I, or STING are often effective at nanomolar to micromolar concentrations. Optimized RIG-I agonist, such as 3p10LG9, exhibits EC_50_ values in the low nanomolar range in primary human skin antigen-presenting cells [91], whereas small molecules such as KIN1400 or STING agonists such as DSDP suppress DENV and ZIKV replication at micromolar concentrations, generally well below levels associated with overt cytotoxicity in vitro [93,95]. Most agonists are maximally effective when used prophylactically or immediately after exposure, as shown for M8 [92]. However, some compounds, like KIN1400, maintain antiviral activity within a limited therapeutic window when administered within a few hours of infection [95].

Furthermore, clinical feasibility is strongly influenced by delivery and bioavailability. Ex vivo studies revealed that RNA-based agonists, such as 3p10LG9, have limited cellular uptake in the absence of dedicated delivery systems. This highlights the importance of developing formulations that facilitate targeted and controlled activation of the innate immune response in relevant cell types [91].

## 5. Conclusions

**Figure 5:**
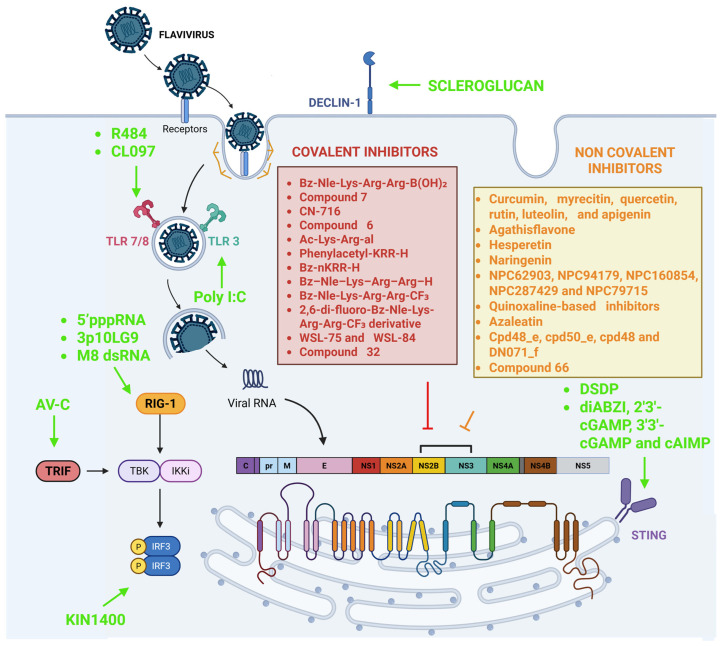
Schematic representation of compounds and pathways involved in the inhibition of Flavivirus replication. The scheme includes immune agonists and NS2B–NS3 protease inhibitors, both covalent and non-covalent. Green arrows indicate the immune agonists and the distinct molecular targets they act on. The red and orange boxes, respectively, group all covalent and non-covalent inhibitors directed against the viral NS2B–NS3 protease. (Created in https://www.biorender.com).

The NS2B–NS3 protease has emerged as one of the most compelling antiviral targets among flaviviruses, and diverse strategies have been developed to block its activity. As summarized in Figure 5, current approaches include both direct-acting protease inhibitors, including covalent and non-covalent agents, and host-directed strategies, such as immune agonists that modulate cellular pathways critical for viral replication. This integrated overview highlights how distinct molecules converge on complementary mechanisms to suppress flaviviral infection.

Peptide-based covalent inhibitors, including boronic acids, aldehydes, TFMK, phenoxymethylphenyl derivatives, and α-ketoamide, have demonstrated remarkable enzymatic potency, though their translation remains limited by bioavailability and metabolic stability. They are highly susceptible to proteolytic degradation and often exhibit poor pharmacokinetic and pharmacodynamic properties, which compromise their bioavailability and limit their therapeutic use [29,97]. To address these challenges, designing modified peptide inhibitors with intrinsic membrane permeability has emerged as a promising strategy. These molecules consist of alkylated amphiphilic peptide heads containing lysines, linked to hydrophobic tails that promote micellar self-assembly. Their supramolecular architecture enhances proteolytic resistance, facilitates membrane penetration, and enables multivalent interactions with target proteases. In particular, it was reported that this Gly-containing geminoid exhibits greater flexibility, allowing it to interact with both the substrate-binding and allosteric sites of the NS2B–NS3 protease complex [98].

An additional strategy to improve bioavailability relies on nanoparticle-based delivery systems. Peptide inhibitors have been shown to cross membrane barriers efficiently when displayed on dextran nanocarriers containing a limited number of cell-penetrating peptide moieties [99,100]. In this context, peptidomimetic inhibitors and cell-penetrating peptides were conjugated to dextran, producing well-defined nanoparticles that acted as potent inhibitors of flaviviral proteases [101]. This configuration enhances proteolytic stability, cellular uptake, and local inhibitor concentration, thereby substantially increasing biological activity. Despite these advances, non-peptidic or peptidomimetic inhibitors have not progressed to clinical development due to limitations in drug metabolism and pharmacokinetics. In this context, the concept of drug repurposing/repositioning is gaining increasing attention, offering the advantage of pre-characterized safety profiles.

Compounds such as temoporfin, used for the treatment of squamous cell carcinoma of the head and neck [102], and niclosamide and nitazoxanide, both FDA-approved anthelmintic drugs [103], have been identified as potent NS2B–NS3 protease inhibitors [104]. Similar results were obtained from Bcl-2 inhibitors (ABT263, ABT737, AT101, TW37) [105], and policresulen [106], a clinical drug used for antimicrobial applications in gynecology, demonstrating antiviral activity with improved bioavailability and established safety. Among the compounds discussed, the most promising candidates for overcoming translational barriers are non-peptidic small-molecule inhibitors and repurposed drugs. The phenoxymethylphenyl derivatives and the α-ketoamide combine a reversible covalent mechanism with nanomolar enzymatic potency and demonstrated cellular antiviral activity, making them suitable for further preclinical optimization [43,44].

Repurposed drugs represent the most promising candidates because of their safety and pharmacokinetic profiles, which may accelerate progression toward in vivo and clinical evaluation [102,103,104,105,106].

These findings highlight the potential of combining rational inhibitor design with repurposing strategies and advanced delivery systems to develop effective therapeutics against dengue and other flaviviral infections.

## Figures and Tables

**Table 2:** Non-covalent inhibitors of NS2B–NS3 protease.

Inhibitor	Type of Inhibitor	Virus Target	Action Site	References
Curcumin	Non-competitive allosteric inhibitor	DENV-2	Allosteric pocket (NS2B–NS3 interface)	[51]
Myricetin	Non-competitive allosteric inhibitor	DENV-2	Allosteric sites AS1-AS2 (NS2B–NS3 interface)	[52]
Curcumin, myricetin, quercetin, rutin, luteolin, and apigenin	Non-competitive allosteric inhibitor	ZIKV	Allosteric conformational site	[53]
Agathisflavone	Non-competitive allosteric inhibitor	DENV-2/ DENV-3	Allosteric pocket adjacent to the catalytic site (NS2B–NS3 interface)	[54]
Hesperidin	Allosteric inhibitor	ZIKV	Allosteric site of NS2B–NS3 protease	[55]
Naringenin	Allosteric inhibitor	ZIKV	Allosteric site of NS2B–NS3 protease	[56]
NPC62903, NPC94179, NPC160854, NPC287429 and NPC79715	Allosteric modulators	DENV	Allosteric pocket adjacent to the catalytic site (NS2B–NS3 interface)	[57]
Quinoxaline-based inhibitors	Allosteric inhibitors	DENV/ ZIKV	Allosteric pocket (NS2B–NS3 interface)	[58]
Azaleatin	Allosteric inhibitor	DENV	Lateral allosteric pocket	[59]
Cpd48_e, cpd50_e, cpd48 and DN071_f	Allosteric inhibitors	DENV/ ZIKV	Allosteric pocket (NS2B–NS3 interface)	[60]
Compound **66**	Non-competitive inhibitor	DENV/ZIKV	Not defined	[61]

## Data Availability

No new data were created or analyzed in this study.

## Abbreviations

The following abbreviations are used in this manuscript:

**Abbreviation**	**Full Name**
5′pppRNA	5′-triphosphate RNA
A172	Human glioblastoma cell line
A549	Human lung carcinoma epithelial cell line
Ala	Alanine
Arg	Arginine
AS1/2	Allosteric site1/2
Asp	Aspartic acid
AV-C	1-(2-fluorophenyl)-2-(5-isopropyl-1,3,4-thiadiazol-2-yl)-1,2-dihydrochromeno[2,3-c]pyrrole-3,9-dione
Bz-nKRR-H	Benzoyl-norleucine-Lys-Arg-Arg-aldehyde
BHK-21	Baby hamster kidney-21 cells
C	Capsid
Catalytic triad	(Ser-His-Asp)
CARD–CARD	Caspase activation and recruitment domain
CC_50_	50% cytotoxic concentration
CDNs	Cyclic dinucleotides
cGAMP	Cyclic GMP–AMP
cGAS	Cyclic GMP–AMP synthase
CLRs	C-type lectin receptors
CN-716	Boronic peptide CN-716 inhibitor
Compound **32**	α-Ketoamide inhibitor
Compound **6**	Boroleucine-derived covalent inhibitor
Compound **7**	Boronic acid peptide inhibitor
CRISPR/Cas9	Clustered Regularly Interspaced Short Palindromic Repeats/CRISPR-associated protein 9
CYD	Chimeric yellow fever–dengue
CYD-TDV	Chimeric yellow fever–dengue tetravalent dengue vaccine (Dengvaxia)
Cys	Cysteine
DENV	Dengue virus
DENV-1	Dengue virus serotype 1
DENV-2	Dengue virus serotype 2
DENV-3	Dengue virus serotype 3
DENV-2 PDK-53	Dengue virus serotype 2 attenuated strain PDK-53
DSDP	Dispiro-diketopiperazine STING agonist
dsRNA	Double-stranded RNA
E	Envelope protein
EC_50_	Half-maximal effective concentratio
EC_68_	68% effective concentration
EMA	European Medicines Agency
ER	Endoplasmic reticulum
FDA	Food and Drug Administration
GAF	Gamma-activated factor
GAS	Gamma-activated sequences
Gln	Glutamine
Glu	Glutamic acid
Gly	Glycine
HepG2	Human hepatoma cells
HFF-1	Human foreskin fibroblasts
His	Histidine
hmdDCs	Human monocyte-derived dendritic cells
HST	Hesperidin
Huh7	Human hepatoma cell line
Huh7.5	Human hepatoma cell line
IC_50_	Half-maximal inhibitory concentration
IC_90_	90% inhibitory concentration
IFIT1	Interferon-induced protein with tetratricopeptide 1
IFIT2	Interferon-induced protein with tetratricopeptide 2
IFITM1	Interferon-induced transmembrane protein 1
IFN-I/ IFN-α/IFN-β	Type I interferons
IFN-II/IFN-γ	Type II interferons
IFN-III/IFN-λ	Type III interferons
IFNAR1	Interferon-alpha/beta receptor 1
IFNAR2	Interferon-alpha/beta receptor 2
IFNGR1	Interferon-gamma receptor 1
IFNGR2	Interferon-gamma receptor 2
IFNLR1	Interferon lambda receptor 1
IFNs	Interferons
IgG1	Immunoglobulin G subclass 1
IgG2a/c	Immunoglobulin G subclass 2a and 2c
IgG2c	Immunoglobulin G subclass 2c
IKK	IκB kinase
IL-10R2	Interleukin-10 receptor 2
IL-1β	Interleukin-1β
IL-2	Interleukin-2
IL-28A/B	Interleukin-28A/B
IL-29	Interleukin-29
IL-6	Interleukin-6
Ile	Isoleucine
IRAK 1/4	Interleukin-1 receptor-associated kinase 1/4
IRF3	Interferon regulatory factor 3
IRF7	Interferon regulatory factor 7
IRF9	Interferon regulatory factor 9
IRFs	Interferon regulatory factors
ISG15	Interferon-stimulated gene 15
ISG54	Interferon-stimulated gene 54 (IFIT2)
ISG56	Interferon-stimulated gene 56 (IFIT1)
ISGF3	Interferon-stimulated gene factor 3
ISGs	Interferon-stimulated genes
ISREs	Interferon-stimulated response elements
JAK1	Janus kinase 1
JAK2	Janus kinase 2
JAK-STAT	Janus kinase-signal transducer and activator of transcription pathway
Ki	Inhibition constant
Ki_ii	Second-order inhibition constant (slow-binding inhibition constant)
Kᴰ	Dissociation constant
LBHB	Low-barrier hydrogen bond
LBHBs	Low-barrier hydrogen bonds
Leu	Leucine
Lys	Lysine
MAVS	Mitochondrial antiviral signaling protein
MD	Molecular dynamics
MDA5	Melanoma differentiation-associated protein 5
MEK/ERK	Mitogen-Activated Protein Kinase/Extracellular signal-Regulated Kinase
Met	Methionine
MX1	Myxovirus resistance protein 1
MX2	Myxovirus resistance protein 2
MyD88	Myeloid differentiation primary response 88
NAR	Naringenin
NF-κB	Nuclear factor kappa-B
NK	Natural killer cell
Nle	Norleucine
NLRs	NOD-like receptors
NMR	Nuclear magnetic resonance
NPASS	Natural Product Activity and Species Source
NS	Nonstructural proteins
NS2B–NS3	Nonstructural 2B–3 protease
NTPase	Nucleoside-triphosphatase
OAS3	Oligoadenylate synthetase 3
ORF	Open reading frame
P1-P4 side chain	Substrate residues on the N-terminal side of the scissile peptide bond (following Schechter and Berger nomenclature)
p38 MAPK	p38 mitogen-activated protein kinase
PAMPs	Pathogen-associated molecular patterns
PBMCs	Peripheral blood mononuclear cells
Phe	Phenylalanine
PKR	Protein Kinase R
poly(I:C)	Polyinosinic:polycytidylic acid
prM	Pre-membrane protein
prM/M	Premembrane/membrane protein
Pro	Proline
PRRs	Pattern-recognition receptors
R848	Resiquimod
RIG-I	Retinoic acid-inducible gene I
RLRs	RIG-I-like receptors
RSAD2	Viperin
S1–S4	Protease subsites that accommodate the P1–P4 substrate residues (following Schechter and Berger nomenclature)
SAR	Structure–Activity Relationship
Ser	Serine
SI	Selectivity index
ssRNA	Single-stranded RNA
STAT1	Cytoplasmic transcription factors 1
STAT2	Cytoplasmic transcription factors 2
STING	Stimulator of interferon genes
TAK-003	tetravalent live-attenuated dengue vaccine (Qdenga^®^)
TBK1	TANK-binding kinase 1
TFMK	Trifluoromethyl ketone
THF	Human fibroblasts with extended replicative lifespan
Thr	Threonine
TLR2	Toll-like receptor 2
TLR3	Toll-like receptor 3
TLR4	Toll-like receptor 4
TLR7	Toll-like receptor 7
TLR8	Toll-like receptor 8
TLR9	Toll-like receptor 9
TLRs	Toll-like receptors
TNF-α	Tumor necrosis factor-α
TRAF6	TNF receptor-associated factor 6
TRIF	TIR-domain-containing adapter-inducing interferon-β
Trp	Tryptophan
TV-003/TV-005	Butantan-DV
TYK2	Tyrosine Kinase 2
Tyr	Tyrosine
U937-DC-SIGN	U937 monocytes expressing DC-SIGN
UTR	Untranslated region
Val	Valine
Vero	African green monkey kidney epithelial cell line
Vero E6	African green monkey kidney epithelial cell line E6
WHO	World Health Organization
WNV	West Nile virus
WSL-75	Phenoxymethylphenyl-derived inhibitor WSL-75
WSL-84	Phenoxymethylphenyl-derived inhibitor WSL-84
YFV	Yellow fever virus
ZIKV	Zika virus
Note: Amino acid numbers (e.g., Ser135, His51, Asp75) refer to residue numbering within the NS2B–NS3 protease sequence of the corresponding Flavivirus.	
